# The Inflation Reduction Act and Drug Development: Potential Early Signals of Impact on Post-Approval Clinical Trials

**DOI:** 10.1007/s43441-025-00774-2

**Published:** 2025-04-22

**Authors:** Hanke Zheng, Julie A. Patterson, Jonathan D. Campbell

**Affiliations:** National Pharmaceutical Council, Washington, DC USA

**Keywords:** Inflation reduction act, Drug price negotiation program, Healthcare innovation, Post-approval clinical development, Health policy

## Abstract

**Background:**

The Inflation Reduction Act’s (IRA) Drug Price Negotiation Program (DPNP) may reduce incentives for industry investments in post-approval clinical development. We aimed to explore the IRA’s impact on the initiation of industry-sponsored, post-approval clinical trials.

**Methods:**

Using Citeline’s Trialtrove database (7/2014–8/2024), we conducted an interrupted time series analysis (ITSA) to estimate the IRA’s impact on the initiation of industry-sponsored Phase I-III trials in previously approved drugs, excluding all vaccines and COVID-19 treatments. We conducted an additional ITSA to examine post-IRA changes in government-funded trials, hypothesized to be unaffected by the IRA, and sensitivity analyses to explore potential exogenous confounding factors. Finally, we explored differences in the IRA’s impact on post-approval industry-sponsored clinical trial initiation in small versus large molecule drugs.

**Results:**

Following the IRA’s passage, the average monthly number of industry-sponsored trials on post-approval drugs decreased by 38.4%. The ITSA indicated that the IRA’s passage was associated with an immediate drop of 11.1 industry-sponsored trials (p-value < 0.05) and an additional decrease by 0.9 trials per month (p-value < 0.01). The IRA’s passage was not statistically associated with changes in government-funded trial initiation. Sensitivity analyses supported ITSA findings. Initiation of post-approval industry-sponsored trials decreased by 47.3% and 32.9% for small and large molecule drugs, respectively.

**Conclusions:**

The IRA’s passage was associated with reductions in industry-sponsored, but not government-funded, post-approval trials, with larger reductions for small molecule drugs. These findings provide early evidence supporting concerns around IRA-related reductions in incentives for post-approval clinical development.

## Introduction

The Inflation Reduction Act (IRA) of 2022 established the Medicare Drug Price Negotiation Program (DPNP) for drugs selected by the Centers for Medicare and Medicaid Services (CMS). Drugs are selected from among single-source drugs with the highest total gross spending in Medicare Parts B and D and are first eligible for selection as early as 7 and 11 years, for small molecule and large molecule drugs, respectively, after initial Food and Drug Administration (FDA) approval [1]. The IRA requires CMS to establish “Maximum Fair Prices” (MFPs) for selected drugs, directing the Agency to “develop and use a consistent methodology and process…that aims to achieve the lowest MFP,” which cannot exceed statutorily defined maximum ceiling prices based on a percentage of nonfederal average manufacturer price (Part D drugs) or average sales price (Part B drugs) [1]. 

Because the IRA shortens the timeline towards price erosion of a drug, previously associated with the loss of market exclusivity, the law may shift or reduce incentives for manufacturer investment in research and development (R&D), including post-approval clinical development [2–6]. Studies describing trajectories and timelines of clinical development suggest post-approval clinical trials are often initiated near or after the time at which drugs will, under the IRA, be eligible for DPNP selection [2, 3, 5]. Similar findings have been reported for timelines towards FDA approvals of subsequent indications [2, 3, 5, 6]. The proportion of clinical trials that are initiated and subsequent indications that receive FDA approval near or after DPNP eligibility is reportedly higher for small molecule drugs, including high-spend Medicare drugs [5] and those for the treatment of cardiovascular disease [3] and cancer [2, 6]. 

In addition to research discussing the IRA’s potential impact in the context of the current landscape of clinical development, several studies have aimed to quantify its potential impact through modeling approaches. Several published models have applied assumptions about the impact of changes in revenue on total new drug approvals to estimate reductions in the number of newly approved drugs over the next 15 to 30 years [7–9]. However, limited evidence is yet available detecting early signals of impact of the IRA on research and development. One study reports a 35% decline in overall clinical trial initiation as well as a 70% decline in aggregate investments in small molecule drugs after passage of the IRA [10]. These results are echoed by a recent survey of life sciences venture capital investors, most of whom (76%) believe that the IRA will decrease investments in life sciences startups and interest in small molecule research and development (87%) [11]. 

The goal of this study was to add to the limited existing research directly quantifying early impacts of the IRA on clinical development. Given the depth of past literature on the potential impact of the IRA’s timelines towards DPNP selection and MFP determination on post-approval research, this study focused on post-approval development as a potential early signal of IRA impact. Specifically, this study aimed to explore the early signal of IRA’s impact on the initiation of the industry-funded Phase I-III clinical trials in previously approved drugs.

## Methods

### Data Sources

This study used Citeline’s Trialtrove database to obtain data on the number of clinical trials initiated between 7/2014 and 8/2024. Citeline’s Trialtrove database comprehensively covers registered drug trials, providing detailed and manually curated trial information. In the main analysis, we included post-approval Phase I-III trials that were primarily sponsored by industry, excluding trials for all vaccines and therapeutics for the prevention and treatment of COVID-19 infections. Government-funded trials were identified using the same inclusion and exclusion criteria for comparison, but required post-approval Phase I-III trials that were primarily sponsored by the government. Data were extracted in 01/2025.

### Study Design

In this longitudinal study, we used interrupted time series analysis (ITSA) to estimate the impact of the passage of the IRA on post-approval clinical development with an aggregated monthly number of trials as the outcome measure. ITSA is a quasi-experimental design broadly used in public health and policy analysis to examine the impact of non-randomized interventions [12]. With the IRA passage in 8/2022 serving as the intervention, we defined the pre-IRA period as 7/2014-7/2022, and the post-IRA period as 8/2022-8/2024. Our analysis compared the level (i.e., number of trials) and trend (i.e., number of trials/month) in post-approval Phase I-III clinical trial initiation during the pre- and post-IRA periods to estimate the impact of the IRA’s passage. To further quantify the potential impact of the IRA, we used the pre-IRA trend estimated from the ITSA model to project the expected number of monthly industry-funded trials at the end of the observation period (8/2024), assuming the IRA had not been passed. This projection was then compared with the actual observation and the predicted trial count in 8/2024 based on the full ITSA model that incorporated the IRA-related level and slope changes, respectively. Note the projection may be a conservative one as the pre-IRA decline likely primarily reflects temporary disruptions due to the COVID-pandemic, which may have gradually resolved over time.

For comparison, we conducted an additional ITSA to examine changes in government-funded trials post-IRA. Government-funded trials were selected as a comparator as they were hypothesized to be unaffected by the IRA but sensitive to other potentially confounding, external changes in the clinical development environment, such as shifts in public health priorities, the COVID-19 pandemic, and technological advancements [13]. 

### Scenario Analyses in Industry- and Government-Funded Trials

In addition to the base-case ITSA, a series of scenario analyses across both industry- and government-funded trials were conducted to test the robustness of our findings. First, past research suggests that threats of pharmaceutical price regulation have had negative impacts on firm-level research and development even when proposed legislation has not become law [14]. Accordingly, recognizing that the effects of the IRA may have begun even before its official passage, we conducted an ITSA modeling the event to start in 11/2021, when the United States House of Representatives first proceeded with debate on the IRA (*Scenario 1)* [1]. 

Second, recent research suggests that the COVID-19 pandemic was associated with delayed clinical trial enrollment and timelines [13]. Because our base-case ITSA included time periods before, during, and after the pandemic, we conducted a scenario analysis limiting the time horizon of the ITSA to a period after the pandemic had begun. Specifically, we ran the ITSA from 1/2020 to 8/2024, such that the pre-IRA period (1/2020-7/2022) excluded pre-pandemic clinical trial environment while still capturing potential impacts of COVID-19 on clinical trial initiation in the pre-IRA period *(Scenario 2)*.

Finally, as a supporting analysis, we ran a difference-in-difference model (DiD) by comparing the changes in industry-funded trials before and after the IRA passage (first difference) with those of government-funded trials (second difference), which served as a counterfactual. The underlying assumption was that trials would have changed similarly in the two groups in the absence of the IRA. The DiD model more directly compares industry- and government-funded trials by comparing whether the two groups changed in the same amount and direction around the interruption (i.e., IRA’s passage). However, unlike the ITSA, this model cannot answer questions about the magnitude of the level or trend change following the interruption, and therefore among other limitations the DiD was used as a secondary and supporting analysis.

### Sensitivity Analyses Within Industry-Funded Trials

Beyond the use of government-funded trials as a comparator (ITSA) and counterfactual (DiD) to control for potentially confounding changes during the study period, we conducted further sensitivity analyses to (1) explore the degree to which specific exogenous factors may have impacted industry-funded trials over time and/or the face validity of the base-case ITSA results, and (2) examine whether the impact of the IRA differed by type of drug (small vs. large molecule) and across selected therapeutic areas.

First, the Federal Reserve increased interest rates 11 times from March 2022 to July 2023 [15]. Smaller and earlier-stage biotechnology companies may be more sensitive to both rising and falling interest rates given their impact on net present value models and company valuation [16, 17]. Accordingly, we explored changes in pre-/post-IRA trial initiation in two subgroups of industry sponsors: top 20 and non-top 20 pharmaceutical companies (identified by Citeline based on overall R&D activity and market presence). The goal of this subgroup analysis was to explore the degree to which financial environment changes may have confounded our estimates of the IRA’s effect. We hypothesized that large pharmaceutical firms would be less sensitive in the short run to the interest rate increases occurring amidst IRA passage when compared to smaller biotechnology firms; that is, we hypothesized that large pharmaceutical firms would have lower relative changes in trial investments due to increased interest rates. Notably, potential differences in IRA impact between large pharmaceutical firms and smaller biotechnology firms may have reflected not only differential interest rate sensitivity but also anecdotal evidence that venture capital (VC) investors of biotechnology firms and their limited partners (LPs) may exhibit an increased risk aversion during the IRA period, leading to decreases in new capital [10]. 

Second, it was hypothesized that significant shifts in the therapeutic market-basket dynamics (e.g., sudden increase the number of drugs going off-patent) may have impacted overall trends in the initiation of new clinical trials in post-approval drugs. We obtained and described data from the U.S. Food and Drug Administration on the number of first-approved generics from 2014 to 2023 (2024 data unavailable) to identify any significant shifts in the volume of generic entry over the study period.

Additionally, the IRA introduced distinct timelines for small and large-molecule drugs to become eligible for price negotiation, which others have described as creating a “pill penalty” disincentivizing initial and ongoing clinical development for small molecule drugs [18]. We described pre-/post-IRA changes in small molecule vs. large-molecule drugs to explore potential differences in the impact of the IRA on post-approval clinical development given the differential timeline towards DPNP eligibility. Finally, we analyzed changes in the number of post-approval trials following the IRA in the five largest therapeutic areas, as defined based on the volume of trials in Citeline: oncology, autoimmune/inflammation, central nervous system, metabolic/endocrinology, and cardiovascular diseases. Trials assigned to multiple therapeutic areas were excluded from this subgroup analysis to avoid potential double counting and classification ambiguity.

### Statistical Approach

Descriptive statistics were applied to summarize the monthly average of newly initiated trials before and after IRA passage across the entire study period, and in a more targeted time from the year prior to IRA passage to the most recently available year. In the ITSA, a linear regression was conducted to evaluate IRA-related changes in level (i.e., immediate shift following the IRA’s passage) and slope (i.e., monthly change in trial initiations compared to the estimated counterfactual without the IRA). Although autocorrelation was not detected, we used Newey-West robust standard errors to enhance the reliability of the estimate. The DiD model was fit using a linear regression, incorporating an indicator of industry- vs. government-funded trials and time indicator to account for time-invariant differences and secular trends unrelated to IRA’s passage. Based on both statistical and visual inspection of the parallel trend assumption, the pre-IRA period for the DiD analysis was defined as 1/2020 to 7/2022. Wilcoxon rank sum test was used to assess whether differences in trial initiation were statistically significant after IRA’s passage in the subgroup analyses of (1) top 20 companies vs. non-top 20 companies, (2) small-molecule vs. large-molecule drugs, and (3) five largest therapeutic areas.

## Results

### Study Sample and Descriptive Findings

A total of 15,259 industry-funded trials and 2,095 government-funded trials were identified throughout the study period. The average (SD) number of industry-funded trials initiated per month was 135.8 (23.8) pre-IRA and 83.6 (13.7) post-IRA (38.4% numeric reduction). The average (SD) number of government-funded trials started per month was 18.4 (5.5) pre-IRA and 12.4 (3.6) post-IRA (32.4% numeric reduction). In the more targeted analysis comparing clinical trial initiation from the year prior to IRA passage to the most recently available year, there was a 30.8% numeric reduction in monthly average of industry-funded trials while the government-funded trials within these same time periods generated a 16.9% numeric reduction.

### Interrupted Time Series Analyses

**Fig. 1:**
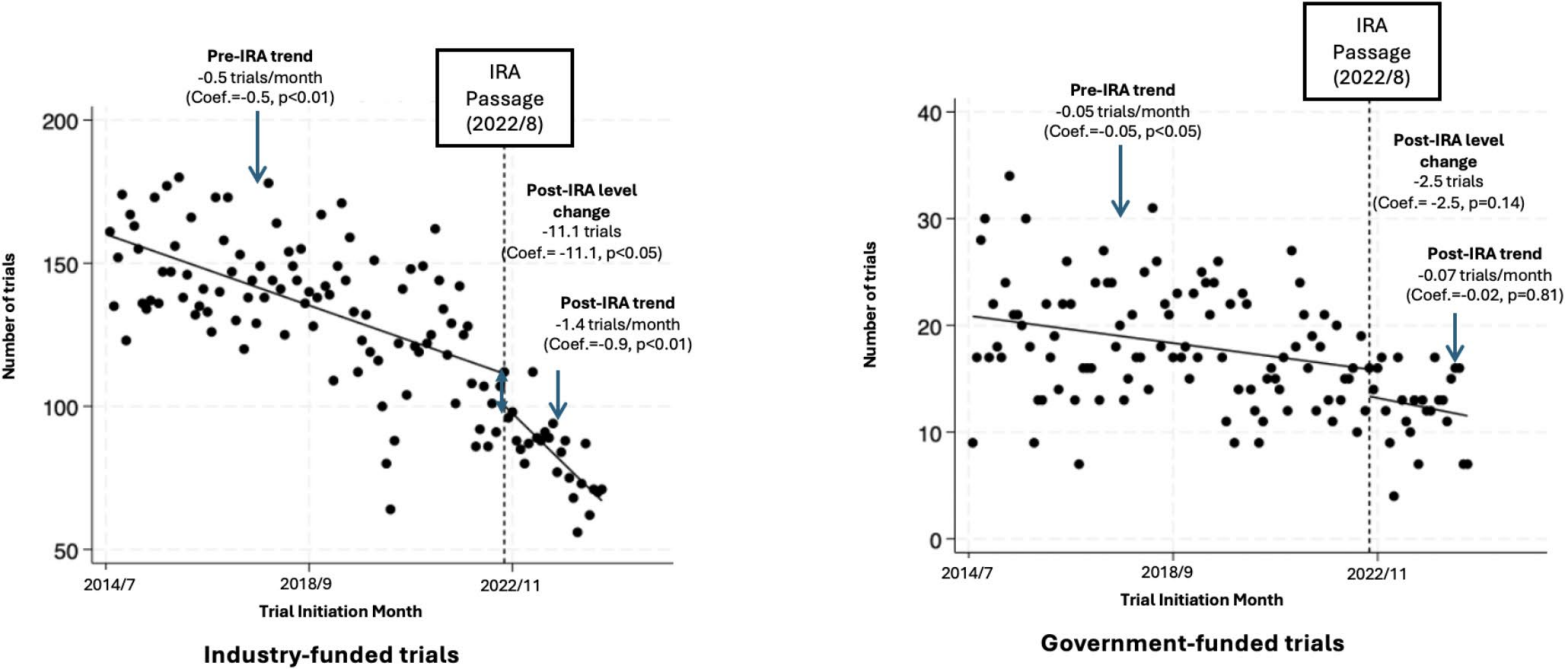
Temporal trends of industry-funded and government-funded clinical trials before and after the IRA’s passage. Abbreviations Used: IRA, Inflation Reduction Act

The ITSA (Fig. 1) indicated that the IRA was associated with an immediate drop of 11.1 industry-funded trials (level change=-11.1, 95% CI: -21.9 to -0.3, p-value < 0.05), and an additional monthly trend decrease by 0.9 industry-funded trials post IRA (slope change=-0.9, 95% CI: -1.4 to -0.4, p-value < 0.01). This post-IRA trend compounded an existing, modest downward trajectory in the pre-IRA period (baseline slope = − 0.5, 95% CI: − 0.6 to − 0.4, *p* < 0.01), resulting in a total average decrease of 1.4 trials per month. Additionally, the projected number of industry-funded trials in 8/2024 was 99, assuming the pre-IRA trend had continued and there was no IRA. In comparison, only 71 trials were actually initiated, reflecting a 28.3% decline associated with the IRA. When incorporating the IRA’s passage, the full ITSA model projected 66.5 trials for 8/2024, suggesting a 32.8% reduction in post-approval trial activity. The ITSA regression that tested for an association between IRA passage and government-funded trials yielded nonsignificant changes in the level (level change =-2.5, 95% CI: -5.9 to 0.8, p-value: 0.14) and trend (slope change =-0.02, 95% CI: -0.2 to 0.2, p-value: 0.81) in monthly clinical trial initiation.

### Scenario Analyses in Industry- and Government-Funded Trials

The negative association between the IRA and industry-funded post-approval clinical trials was robust in scenario analyses of the ITSA model (Table 1). Modeling an earlier IRA interruption to account for a potential earlier impact when the IRA entered in debate (*Scenario 1*), there was an immediate reduction of 14.4 industry-funded trials (level change = -14.4, 95% CI: -26.4 to -2.3, p-value < 0.05) and an additional monthly decrease of 0.8 industry-funded trials associated with the IRA (slope change = − 0.8, 95% CI: -1.2 to -0.4, p-value < 0.01). In *Scenario 2*, we modeled the impact of the IRA on clinical trial initiation in the shorter, post-COVID time. The ITSA of industry-sponsored trials in *Scenario* 2 showed a numeric immediate reduction of 11.4 trials (level change = -11.4, 95% CI: -27.4 to 4.6, p-value: 0.16) and a drop of an additional 1.2 trials per month (slope change = -1.2, 95% CI: -2.2 to -0.1, p-value < 0.05), after the IRA passage. No statistically significant association was observed between IRA passage and changes in the government-funded trials across either modeled scenario.

**Table 1:** Interrupted time series analysis on the impact of the IRA on the initiation of Industry- and Government-Funded clinical trials on previously approved drugs

	Model Components		IRA Effect (Trials per Month)	
	IRA cutoff	Time Frame	Immediate level change	Slope change
**Industry-funded trials**				
Base case	IRA passage	2014/7-2024/8	-11.1^*^	-0.9^**^
Scenario 1	IRA in debate	2014/7-2024/8	-14.4^*^	-0.8^**^
Scenario 2	IRA passage	2020/1-2024/8	-11.4	-1.2^*^
**Government-funded trials**				
Base case	IRA passage	2014/7-2024/8	-2.5	-0.02
Scenario 1	IRA in debate	2014/7-2024/8	-2.3	-0.07
Scenario 2	IRA passage	2020/1-2024/8	-1.9	-0.02

Abbreviations Used: IRA, Inflation Reduction Act; NA, not applicable

^*^ P-value < 0.05; ^**^ P-value < 0.01

Scenario 1 modeled the intervention (IRA) to start in 11/2021, when the United States House of Representatives first proceeded with debate on the IRA.^1^ Scenario 2 analyzed data from 1/2020 to 8/2024, such that the pre-IRA period (1/2020-7/2022) excluded pre-pandemic clinical trial environment while still capturing potential impacts of COVID-19 on clinical trial initiation.^13^

In the DiD model, we found that IRA passage was associated with a statistically significant average reduction of 27.5 industry-funded trials when compared to government-funded trials per month over the study period (DiD Coefficient = -27.5, 95% CI: -37.0 to -18.0, p-value < 0.01).

### Sensitivity Analyses Within Industry-Funded Trials

**Fig. 2:**
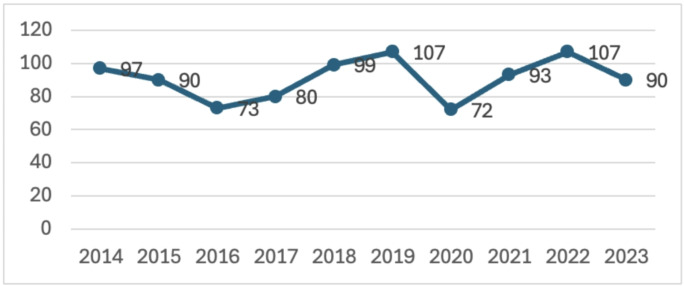
Number of first generic entries per year approved by the Food and Drug Administration, 2014–2023

We explored the potential exogenous effects of interest rate and market basket dynamics, as well as the potential differential impact of the IRA on small molecule drugs, among industry-funded trials (Table 2). In the stratified analysis by trial sponsor size, approximately a third (37.4%) of trials were sponsored by a company among the largest 20 by the overall R&D activity and market presence. After IRA’s passage, there was a reduction by 42.7% in trials sponsored by the top 20 companies (p-value < 0.01) and a decrease by 35.7% of trials sponsored by the non-top 20 companies (p-value < 0.01). Regarding market basket changes, we found that the number of first generic entries remained stable throughout the study period, indicating a stable market dynamic during this time (Fig. 2).

**Table 2:** Number of Post-Approval clinical trials initiated per month before and after the IRA’s passage by subgroups (Within Industry-Funded Trials)

Category	Trial counts (%)	Monthly average (SD) pre-IRA passage	Monthly average (SD) post-IRA passage	Relative difference
Overall	100%	135.8 (23.8)	83.6 (13.7)	-38.4%^*^
Drug Type				
Small molecule^†^	60.6%	83.9 (19.2)	44.2 (9.9)	-47.3%^*^
Large molecule^†^	18.2%	24.4 (6.7)	16.4 (5.3)	-32.9%^*^
Sponsor Type				
Top 20 companies	37.4%	51.3 (14.2)	29.3 (5.9)	-42.7%^*^
Non-top 20 companies	62.6%	84.5 (14.3)	54.3 (10.0)	-35.7%^*^
Therapeutic Area				
Oncology^‡^	36.3%	47.8 (8.6)	36.4 (6.2)	-23.8%^*^
Autoimmune/inflammation^‡^	15.6%	21.1 (5.5)	13.8 (3.3)	-34.7%^*^
Central nervous system^‡^	13.6%	18.7 (6.4)	10.2 (4.2)	-45.5%^*^
Metabolic/endocrinology^‡^	13.1%	18.5 (5.7)	8.2 (4.5)	-55.6%^*^
Cardiovascular diseases^‡^	7.2%	9.9 (3.8)	5.6 (2.6)	-43.1%^*^

Abbreviations Used: IRA, Inflation Reduction Ac; SD, standard deviation

^†^ Trials of which the primary tested drugs involved both small-molecule and large-molecule drugs were excluded in the subgroup analysis, suggesting a sum that is below the Overall total

^‡^ Trials assigned to other therapeutic areas were not included, suggesting a sum that is below the Overall total

^*^ P-value < 0.01

Overall, the primary tested drug was a small molecule in 60.6% of included trials, while 18.2% of included trials tested a large-molecule drug. The remainder of trials (21.2%) were excluded from analyses on small and large molecule drug trials because the tested drug included both types of the drug (e.g., combination therapies). After the passage of the IRA, the average number of post-approval industry-funded trials initiated significantly decreased by 47.3% for small molecule drugs (pre-IRA: 83.9 per month (19.2); post-IRA: 44.2 per month (9.9) p-value < 0.01) and by 32.9% for large-molecule drugs (pre-IRA: 24.4 per month (6.7); post-IRA: 16.4 per month (5.3), p-value < 0.01). Finally, the average number of trials initiated per month declined consistently across all five therapeutic areas after the passage of the IRA, with relative reductions ranging from 23.8% in oncology to 55.6% in metabolic/endocrinology. These differences were statistically significant (p-value < 0.01) in all comparisons,

## Discussion

This study provides early evidence of the IRA’s potential impact on industry investments in post-approval clinical development. Comparing industry-funded trials with government-funded trials, the evidence supports a reduction in industry-funded, post-approval clinical trials associated with IRA passage while not supporting a reduction in government-funded trials. The industry-funded post-approval trial decline associated with the IRA was demonstrated through a broad, representative sample of trials encompassing all therapeutic areas except COVID-19 and vaccines, sourced from validated registries and databases. This early signal of IRA impact is consistent with researcher perspectives that the timelines of the IRA, clinical development, and FDA approvals for subsequent indications suggest that the law will reduce incentives for post-approval research and development [2–6, 10]. While the primary focus of this study was on the impact of the IRA, we acknowledge that there was a small decline in industry-funded post-approval trials during the pre-IRA period. This pre-IRA trend likely reflects the effects of the COVID-19 pandemic, along with broader structural shifts in the biopharmaceutical sector, such as a growing focus on early-phase development and evolving strategies for post-approval research. Although modest, this pre-IRA trend suggests that industry investment in post-approval research may have already been under pressure prior to the policy change that likely further compounded it.

The association between the IRA and a reduction in industry-funded post-approval clinical trials was robust to scenario and sensitivity analyses considering the potential confounding effects of diverse exogeneous factors. First, the negative association between IRA passage and industry-funded trials was not observed in an ITSA with government-funded trials, which were hypothesized to be unaffected by the IRA but sensitive to late impacts of the COVID-19 pandemic and shifts in public health priorities. Secondly, when varying the ITSA model components—accounting for potential early IRA effects when the law was debated or using an exclusively post-COVID time window — the negative association between the IRA and trial initiation remained consistent for industry-funded trials. At the same time, there continued to be no significant effect of the IRA on government-funded trials. These results suggest that the impact of the IRA was over and beyond residual effects of changes in the clinical trial environment following the pandemic [19]. Scenario analyses further supported that the IRA’s impact on industry-sponsored clinical trials may have started as the IRA entered public debate, consistent with early impacts on investment amidst past threats of government price regulation [14]. Finally, the ITSA estimates (e.g. combination of the changes in level and slope) for industry-funded trials align closely with the ‘average treatment effect’ of IRA’s passage as estimated by the DiD model. The DiD model, though constrained by a shorter pre-IRA period than the ITSA, explicitly incorporated government-funded trials as the counterfactual to account for the secular trends unrelated to IRA.

Sensitivity analyses exploring potentially confounding factors specific to industry-funded trials lend additional support to the results’ robustness. We descriptively compared declines in industry-funded post-approval trials by company size, finding a numerically larger percentage reduction in trials sponsored by “Top 20” companies after IRA passage compared with those supported smaller companies. Notably, smaller pharmaceutical and biotechnology companies were likely to be more sensitive to the federal interest rate increases over the observed time period than their larger counterparts [16, 17]. Larger pharmaceutical firms saw larger reductions in trials post-IRA, suggesting that the observed overall decline in post-approval trials was less likely due to financial environment changes and supports concerns about the IRA’s impact. Moreover, the number of first generic entries remained stable throughout the study period, mitigating concerns that observed trends reflected significant changes in the volume of drugs approaching the end of patent or market exclusivity over the study period.

Notably, our findings indicate a larger impact of the IRA on industry-sponsored, post-approval development for small molecule drugs compared with large molecule drugs. The IRA establishes a “clock” from a drug’s initial FDA approval to its eligibility for selection to the DPNP that is shorter for small molecule drugs (i.e., 7 years) than large molecule drugs (i.e., 11 years). The observation that small molecule drugs, faced with a shorter “clock” to government price regulation, have already seen a larger impact on post-approval development than large molecule drugs supports concern of a “pill penalty” as discussed by others [18] and in peer-reviewed literature [2, 3, 5, 6]. Furthermore, we revealed a consistent post-IRA decline in post-approval clinical development across main therapeutic areas, reflecting a potential systemic shift in research and development investment priorities in the post-approval phase.

While we did not specifically investigate other subsets of trials due to sample size limitations, it is important to note that the IRA and CMS’s interpretation of the law in Guidance may disproportionately reduce incentives for post-approval research in specific subgroups of drugs. For example, the IRA statute excludes from selection orphan drugs that are “designated…for only one rare disease or condition…and for which the only approved indication (or indications) is for such disease or condition.” [1] CMS has published its interpretation of the orphan drug exclusion (ODE) provision, maintaining that active orphan designations towards additional rare diseases disqualify drugs from the ODE, even if not yet approved [20]. This interpretation of the ODE has generated concern that the law may disincentivize subsequent indications in orphan drugs, including for serious illnesses with high unmet need [21]. The disincentivizing effect of the IRA on post-approval clinical development has also been discussed as particularly relevant for oncology, where multiple indications are both common and critical in expanding treatment options for patients [2, 6]. Several policy proposals are being discussed to mitigate the potential adverse impacts of the IRA on clinical development, including, but not limited to, (1) delaying DPNP eligibility for new indications, (2) excluding from DPNP eligibility orphan drugs that treat one or more rare conditions, (3) extending the small molecule DPNP eligibility “clock” to align with the timeline for large molecule drugs, and (4) developing a transparent, consistent framework for MFP determination that measures and appreciates the comprehensive value of medicines [22–25]. 

This study has several limitations. First, ITSA models were performed by focusing on how the IRA may impact the outcome measure. While we performed a variety of sensitivity and scenario analyses, including ITSA and DiD approaches to comparing industry-sponsored trials with government-funded trials, we cannot exclude the possibility that unobserved or unmeasured confounding factors may have influenced industry-sponsored post-approval trials during the study period. Additionally, the relatively low number of government-funded trials may limit the variability and reduce the statistical power of the DiD model. Moreover, while Citeline’s Trialtrove sourced the trial data from multiple validated data sources, it may not capture the full landscape of post-approval clinical trials. Future research should explore complementary data sources to enhance the robustness and generalizability of the findings. Another limitation is that the Citeline data could not differentiate voluntary, manufacturer-sponsored post-approval trials from post-marketing requirements (PMRs) and post-marketing commitments (PMCs) that are mandated by or agreed upon with the FDA. Our exclusion of Phase IV trials likely mitigated the number of PMRs and PMCs captured in the data, but the sample may have retained some of these studies. Given regulatory involvement, these trials would likely have been less sensitive to the impact of the IRA, thereby lessening the likelihood of an industry-funded statistically significant result. Furthermore, our analysis focused solely on post-approval trials, which limited our ability to capture the broader impact of the IRA on all types of research and development investments. Although we identified early signals of the IRA’s impact on post-approval clinical development, our time horizon could not capture manufacturer responses to ongoing IRA implementation, including publication of the first MFPs, public explanations of the MFPs, or selection of the second cohort of drugs. Finally, due to data limitations, we could not assess the potential implications of discontinued trials on patient outcomes and public health from a broader societal perspective, leading to further uncertainty regarding the impact of potentially discontinued trials on patient health. Future research is needed to evaluate long-term and ongoing effects of the law on clinical development, as well as its impact on patient welfare and public health.

## Conclusion

This research contributes to the broader objective of measuring the holistic impacts of the IRA, including the potential to reduce incentives for industry-funded post-approval clinical trials. The findings support concerns around IRA-related changes to clinical development by demonstrating a persistent reduction in industry-funded post-approval clinical trials following IRA passage. This analysis, which includes the COVID-19 pandemic in the pre-period but removes COVID-19 therapies throughout, provides evidence that, in contrast to government trial sponsors, manufacturers may already be responding to reduced incentives for investments in post-approval clinical research post-IRA, particularly for small molecule therapies. Future research should continue to monitor the long-term impact of the IRA, including in subgroups of drugs that may be disproportionately impacted.

## Data Availability

No datasets were generated or analysed during the current study.

## References

[1] 117th Congress. (2021–2022). H.R.5376 - Inflation Reduction Act of 2022. https://www.congress.gov/bill/117th-congress/house-bill/5376. Published 2021. Accessed2024.

[2] Grabowski H, DiMasi JA, Long G. Postapproval innovation for oncology drugs and the inflation reduction act. Health Aff (Millwood). 2024;43(10):1400–9. 39374451 10.1377/hlthaff.2024.00202

[3] Grabowski H, Long G. Post-approval indications and clinical trials for cardiovascular drugs: some implications of the US inflation reduction act. J Med Econ. 2024;27(1):463–72. 38419523 10.1080/13696998.2024.2323903

[4] O’Brien JM, Motyka J, Patterson JA. How the IRA could delay pharmaceutical launches, reduce indications, and chill evidence generation. Health Affairs Forefront; 2023.

[5] Patterson J, Motyka J, O’Brien JM. Unintended consequences of the inflation reduction act: clinical development toward subsequent indications. Am J Manag Care. 2024;30(2):82–6. 38381543 10.37765/ajmc.2024.89495

[6] Patterson JA, Motyka J, Salih R, Nordyke R, O’Brien JM, Campbell JD. Subsequent indications in oncology drugs: pathways, timelines, and the inflation reduction act. Ther Innov Regul Sci. 2025;59(1):102–11. 39369117 10.1007/s43441-024-00706-6 PMC11706854

[7] Philipson TJ, Durie T, The Impact of HR 5376 on Biopharmaceutical Innovation and Patient Health. https://cpb-us-w2.wpmucdn.com/voices.uchicago.edu/dist/d/3128/files/2021/08/Issue-Brief-Drug-Pricing-in-HR-5376-11.30.pdf. Published 2021. Accessed2024.

[8] Office CB. Effects of Drug Price Negotiation Stemming From Title 1 of H.R. 3, the Lower Drug Costs Now Act of 2019, on Spending and Revenues Related to Part D of Medicare Letter to House Committee on Energy and Commerce. https://www.cbo.gov/system/files/2019-10/hr3ltr.pdf. Published 2019. Accessed.

[9] Vogel M, Kakani P, Chandra A, Conti RM. Medicare price negotiation and pharmaceutical innovation following the inflation reduction act. Nat Biotechnol. 2024;42(3):406–12. 38297186 10.1038/s41587-023-02096-w

[10] O’Loughlin G, Askeland M, Gassull D, Bowen HP. The Inflation Reduction Act’s Impact upon Early-stage Venture Capital Investments. https://www.medrxiv.org/content/medrxiv/early/2025/01/07/2025.01.07.25320113.full.pdf. Published 2025. Accessed2025. 10.1007/s43441-025-00773-3 PMC12181096 40223014

[11] Coalition I. Biotech’s Growth Stifled by Policy Missteps. 2025.

[12] Turner SL, Karahalios A, Forbes AB, Taljaard M, Grimshaw JM, McKenzie JE. Comparison of six statistical methods for interrupted time series studies: empirical evaluation of 190 published series. BMC Med Res Methodol. 2021;21(1):134. 34174809 10.1186/s12874-021-01306-w PMC8235830

[13] Sathian B, Asim M, Banerjee I, et al. Impact of COVID-19 on clinical trials and clinical research: A systematic review. Nepal J Epidemiol. 2020;10(3):878–87. 33042591 10.3126/nje.v10i3.31622 PMC7538012

[14] Golec J, Hegde S, Vernon JA. Pharmaceutical R&D spending and threats of price regulation. J Financ Quant Anal. 2010;45(1):239–64.

[15] Rodini L. A timeline of the Fed’s ‘22–‘23 rate hikes & what caused them. https://www.thestreet.com/fed/fed-rate-hikes-2022-2023-timeline-discussion. Published 2024. Accessed2024.

[16] Zamecnik A. Money moves: How the biotech market is weathering inflationary storms. https://www.pharmaceutical-technology.com/features/money-moves-how-the-biotech-market-is-weathering-inflationary-storms/. Published 2023. Accessed2024.

[17] MOOMOO Technologies Inc. Rate cuts on the horizon: How to invest in interest-rate sensitive biotech companies. https://www.moomoo.com/us/learn/detail-rate-cuts-on-the-horizon-how-to-invest-in-interest-rate-sensitive-biotech-companies-117351-240896039. Published 2024. Accessed2024.

[18] Reilly L. Published. The IRA’s pill penalty threatens the future of oncology R&D. PhRMA. https://phrma.org/Blog/The-IRAs-pill-penalty-threatens-the-future-of-oncology-R-and-D. 2023. Accessed2024.

[19] Gelfand JMHB. Clinical research after COVID-19: embracing a new normal. J Invest Dermatol. 2021;141(3):481–3. 32890628 10.1016/j.jid.2020.08.004 PMC7466954

[20] Services CfMaM. Medicare Drug Price Negotiation Program: Final Guidance, Implementation of Sects. 1191–1198 of the Social Security Act for Initial Price Applicability Year 2027 and Manufacturer Effectuation of the Maximum Fair Price in 2026 and 2027 In:2024.

[21] Chambers J, Clifford K, Enright D. Follow-On indications for orphan drugs related to the inflation reduction act. JAMA Netw Open. 2023;6(8):e2329006. 37581890 10.1001/jamanetworkopen.2023.29006 PMC10427936

[22] Goldman D, Grogan J, Lakdawalla D et al. Mitigating the inflation reduction act’s adverse impacts on the prescription drug market. USC Schaeffer Center White Paper Series. https://healthpolicy.usc.edu/research/mitigating-the-inflation-reduction-acts-potential-adverse-impacts-on-the-prescription-drug-market/?utm_source=chatgpt.com. Published 2023. Accessed2024.

[23] National Pharmaceutical Council. NPC Submits Comments to CMS on Draft Guidance for the Medicare Drug Price Negotiation Program for 2027. https://www.npcnow.org/resources/npc-submits-comments-cms-draft-guidance-medicare-drug-price-negotiation-program-2027. Published 2024. Accessed2024.

[24] 118th Congress. (2023–2024). H.R.7174 - To amend title XI of the Social Security Act to equalize the negotiation period between small-molecule and biologic candidates under the Drug Price Negotiation Program. https://www.congress.gov/bill/118th-congress/house-bill/7174. Published 2024. Accessed2024.

[25] 118th Congress. (2023–2024). H.R.5539 - ORPHAN Cures Act. https://www.congress.gov/bill/118th-congress/house-bill/5539. Published 2023. Accessed2024.

